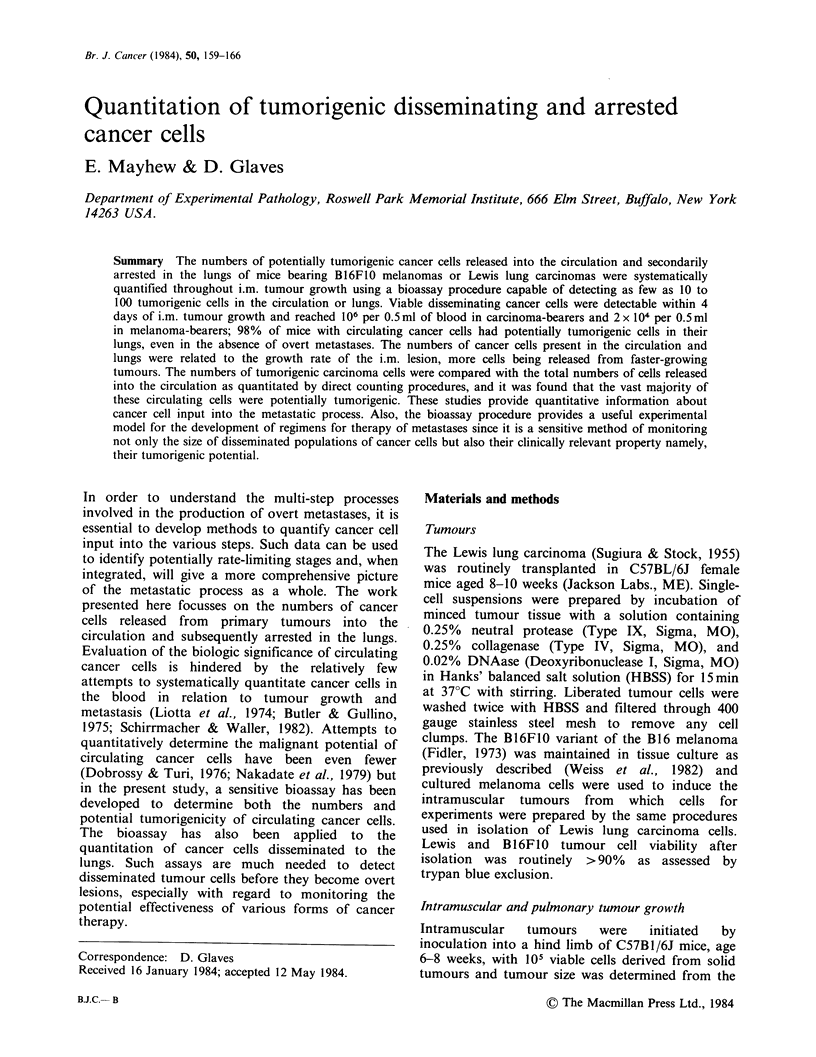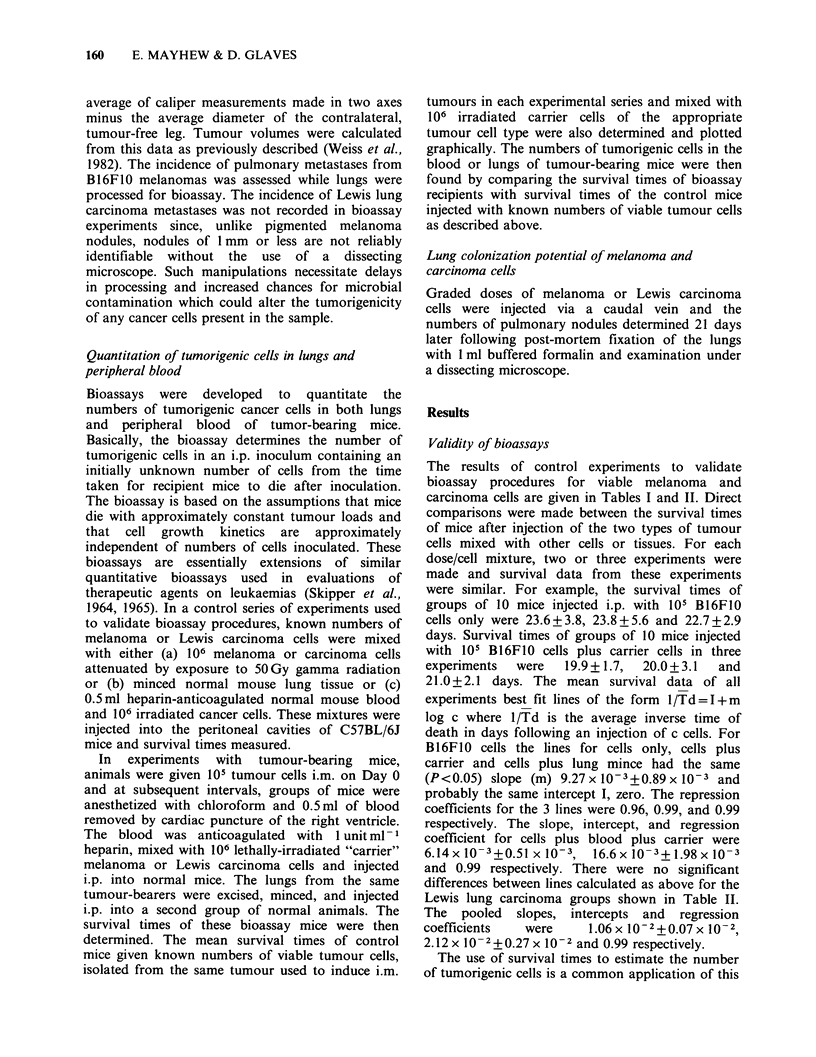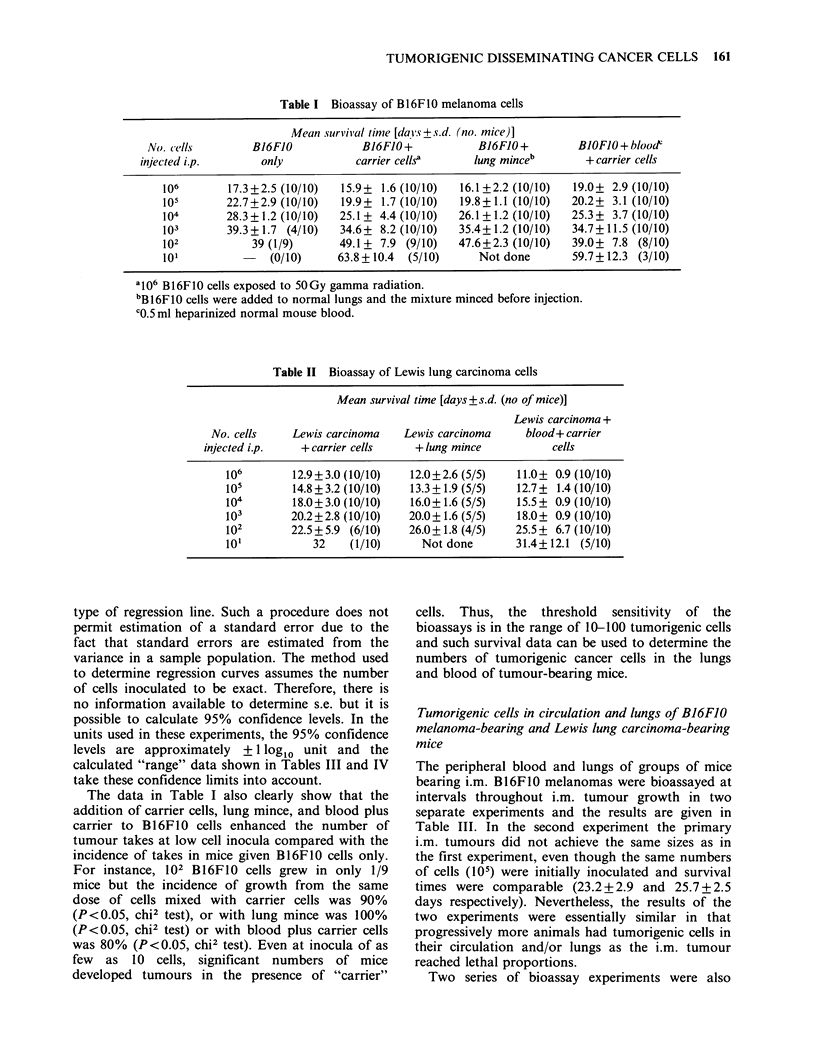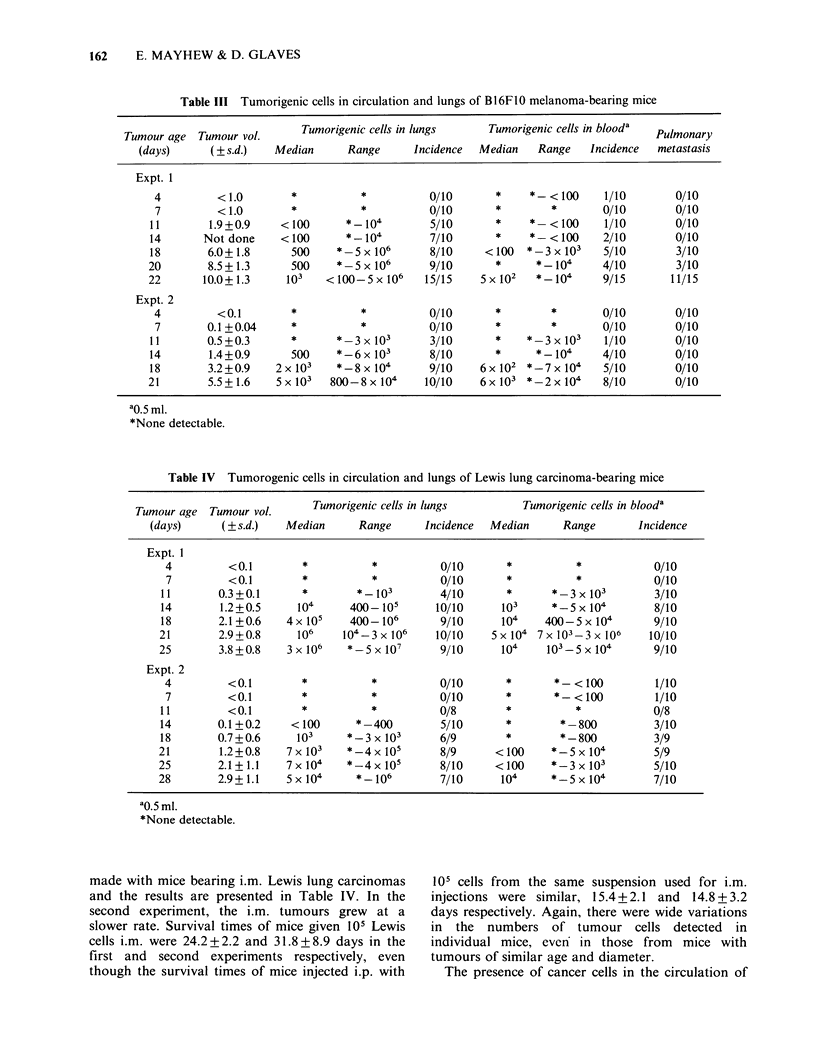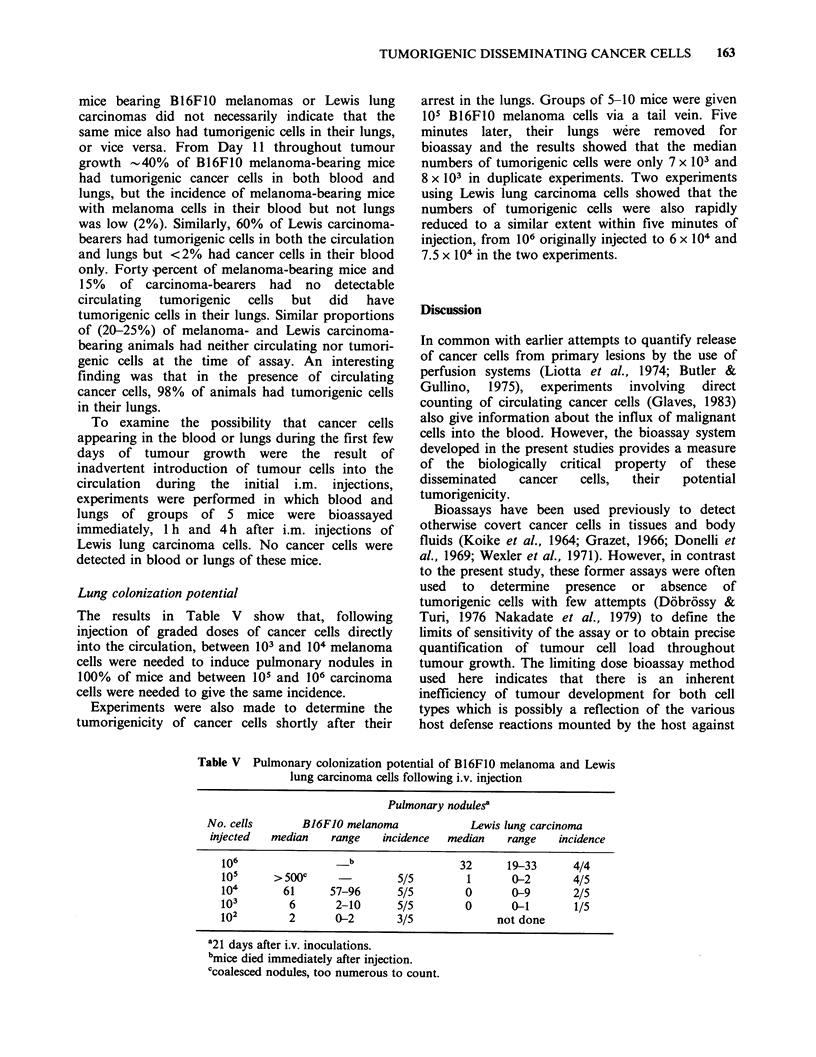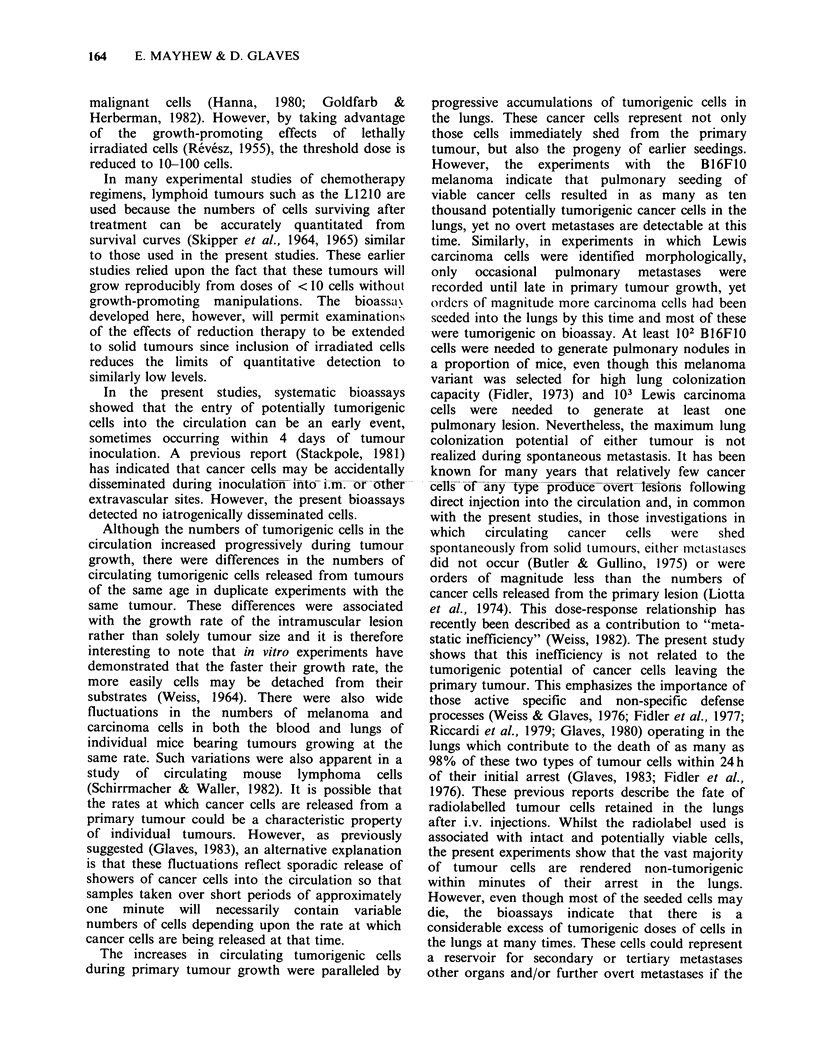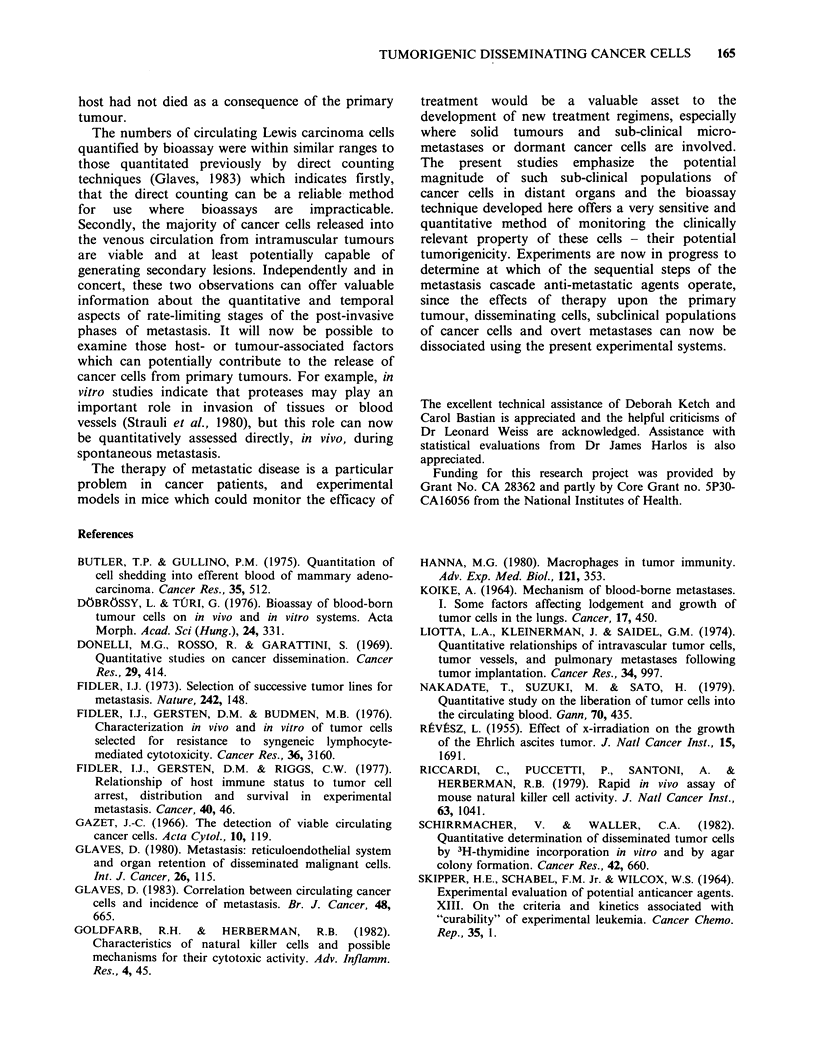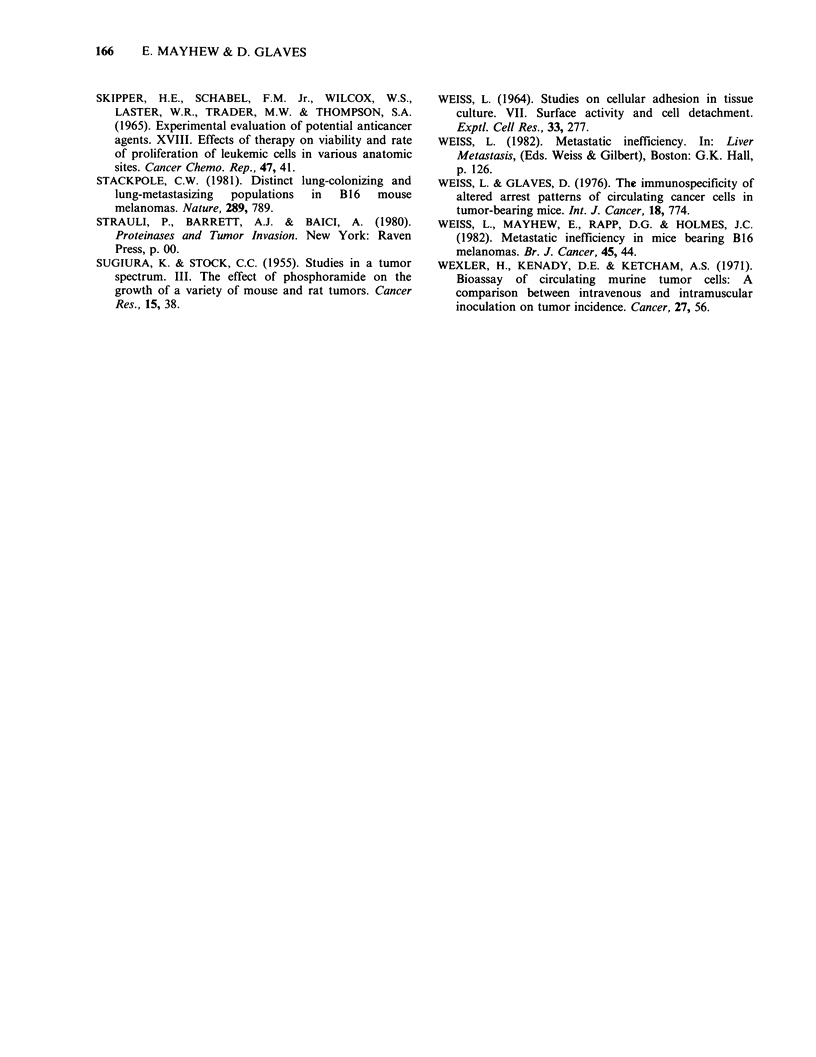# Quantitation of tumorigenic disseminating and arrested cancer cells.

**DOI:** 10.1038/bjc.1984.158

**Published:** 1984-08

**Authors:** E. Mayhew, D. Glaves

## Abstract

The numbers of potentially tumorigenic cancer cells released into the circulation and secondarily arrested in the lungs of mice bearing B16F10 melanomas or Lewis lung carcinomas were systematically quantified throughout i.m. tumour growth using a bioassay procedure capable of detecting as few as 10 to 100 tumorigenic cells in the circulation or lungs. Viable disseminating cancer cells were detectable within 4 days of i.m. tumour growth and reached 10(6) per 0.5 ml of blood in carcinoma-bearers and 2 X 10(4) per 0.5 ml in melanoma-bearers; 98% of mice with circulating cancer cells had potentially tumorigenic cells in their lungs, even in the absence of overt metastases. The numbers of cancer cells present in the circulation and lungs were related to the growth rate of the i.m. lesion, more cells being released from faster-growing tumours. The numbers of tumorigenic carcinoma cells were compared with the total numbers of cells released into the circulation as quantitated by direct counting procedures, and it was found that the vast majority of these circulating cells were potentially tumorigenic. These studies provide quantitative information about cancer cell input into the metastatic process. Also, the bioassay procedure provides a useful experimental model for the development of regimens for therapy of metastases since it is a sensitive method of monitoring not only the size of disseminated populations of cancer cells but also their clinically relevant property namely, their tumorigenic potential.


*Br. J. Cancer* (1984), **50**, 159–166

### Quantitation of tumorigenic disseminating and arrested cancer cells

E. Mayhew & D. Glaves

*Department of Experimental Pathology, Roswell Park Memorial Institute, 666 Elm Street, Buffalo, New York 14263 USA.*

**Summary** The numbers of potentially tumorigenic cancer cells released into the circulation and secondarily arrested in the lungs of mice bearing B16F10 melanomas or Lewis lung carcinomas were systematically quantified throughout i.m. tumour growth using a bioassay procedure capable of detecting as few as 10 to 100 tumorigenic cells in the circulation or lungs. Viable disseminating cancer cells were detectable within 4 days of i.m. tumour growth and reached $10^6$ per 0.5 ml of blood in carcinoma-bearers and $2 \times 10^4$ per 0.5 ml in melanoma-bearers; 98% of mice with circulating cancer cells had potentially tumorigenic cells in their lungs, even in the absence of overt metastases. The numbers of cancer cells present in the circulation and lungs were related to the growth rate of the i.m. lesion, more cells being released from faster-growing tumours. The numbers of tumorigenic carcinoma cells were compared with the total numbers of cells released into the circulation as quantitated by direct counting procedures, and it was found that the vast majority of these circulating cells were potentially tumorigenic. These studies provide quantitative information about cancer cell input into the metastatic process. Also, the bioassay procedure provides a useful experimental model for the development of regimens for therapy of metastases since it is a sensitive method of monitoring not only the size of disseminated populations of cancer cells but also their clinically relevant property namely, their tumorigenic potential.

In order to understand the multi-step processes involved in the production of overt metastases, it is essential to develop methods to quantify cancer cell input into the various steps. Such data can be used to identify potentially rate-limiting stages and, when integrated, will give a more comprehensive picture of the metastatic process as a whole. The work presented here focusses on the numbers of cancer cells released from primary tumours into the circulation and subsequently arrested in the lungs. Evaluation of the biologic significance of circulating cancer cells is hindered by the relatively few attempts to systematically quantitate cancer cells in the blood in relation to tumour growth and metastasis (Liotta *et al.*, 1974; Butler & Gullino, 1975; Schirrmacher & Waller, 1982). Attempts to quantitatively determine the malignant potential of circulating cancer cells have been even fewer (Dobrossy & Turi, 1976; Nakadate *et al.*, 1979) but in the present study, a sensitive bioassay has been developed to determine both the numbers and potential tumorigenicity of circulating cancer cells. The bioassay has also been applied to the quantitation of cancer cells disseminated to the lungs. Such assays are much needed to detect disseminated tumour cells before they become overt lesions, especially with regard to monitoring the potential effectiveness of various forms of cancer therapy.

Correspondence: D. Glaves
Received 16 January 1984; accepted 12 May 1984.

### Materials and methods

### *Tumours*

The Lewis lung carcinoma (Sugiura & Stock, 1955) was routinely transplanted in C57BL/6J female mice aged 8–10 weeks (Jackson Labs., ME). Single-cell suspensions were prepared by incubation of minced tumour tissue with a solution containing 0.25% neutral protease (Type IX, Sigma, MO), 0.25% collagenase (Type IV, Sigma, MO), and 0.02% DNAase (Deoxyribonuclease I, Sigma, MO) in Hanks' balanced salt solution (HBSS) for 15 min at 37°C with stirring. Liberated tumour cells were washed twice with HBSS and filtered through 400 gauge stainless steel mesh to remove any cell clumps. The B16F10 variant of the B16 melanoma (Fidler, 1973) was maintained in tissue culture as previously described (Weiss *et al.*, 1982) and cultured melanoma cells were used to induce the intramuscular tumours from which cells for experiments were prepared by the same procedures used in isolation of Lewis lung carcinoma cells. Lewis and B16F10 tumour cell viability after isolation was routinely >90% as assessed by trypan blue exclusion.

### *Intramuscular and pulmonary tumour growth*

Intramuscular tumours were initiated by inoculation into a hind limb of C57B1/6J mice, age 6–8 weeks, with $10^5$ viable cells derived from solid tumours and tumour size was determined from the

© The Macmillan Press Ltd., 1984

average of caliper measurements made in two axes minus the average diameter of the contralateral, tumour-free leg. Tumour volumes were calculated from this data as previously described (Weiss *et al.*, 1982). The incidence of pulmonary metastases from B16F10 melanomas was assessed while lungs were processed for bioassay. The incidence of Lewis lung carcinoma metastases was not recorded in bioassay experiments since, unlike pigmented melanoma nodules, nodules of 1 mm or less are not reliably identifiable without the use of a dissecting microscope. Such manipulations necessitate delays in processing and increased chances for microbial contamination which could alter the tumorigenicity of any cancer cells present in the sample.

*Quantitation of tumorigenic cells in lungs and peripheral blood*

Bioassays were developed to quantitate the numbers of tumorigenic cancer cells in both lungs and peripheral blood of tumor-bearing mice. Basically, the bioassay determines the number of tumorigenic cells in an i.p. inoculum containing an initially unknown number of cells from the time taken for recipient mice to die after inoculation. The bioassay is based on the assumptions that mice die with approximately constant tumour loads and that cell growth kinetics are approximately independent of numbers of cells inoculated. These bioassays are essentially extensions of similar quantitative bioassays used in evaluations of therapeutic agents on leukaemias (Skipper *et al.*, 1964, 1965). In a control series of experiments used to validate bioassay procedures, known numbers of melanoma or Lewis carcinoma cells were mixed with either (a) $10^6$ melanoma or carcinoma cells attenuated by exposure to 50 Gy gamma radiation or (b) minced normal mouse lung tissue or (c) 0.5 ml heparin-anticoagulated normal mouse blood and $10^6$ irradiated cancer cells. These mixtures were injected into the peritoneal cavities of C57BL/6J mice and survival times measured.

In experiments with tumour-bearing mice, animals were given $10^5$ tumour cells i.m. on Day 0 and at subsequent intervals, groups of mice were anesthetized with chloroform and 0.5 ml of blood removed by cardiac puncture of the right ventricle. The blood was anticoagulated with 1 unit ml$^{-1}$ heparin, mixed with $10^6$ lethally-irradiated "carrier" melanoma or Lewis carcinoma cells and injected i.p. into normal mice. The lungs from the same tumour-bearers were excised, minced, and injected i.p. into a second group of normal animals. The survival times of these bioassay mice were then determined. The mean survival times of control mice given known numbers of viable tumour cells, isolated from the same tumour used to induce i.m. tumours in each experimental series and mixed with $10^6$ irradiated carrier cells of the appropriate tumour cell type were also determined and plotted graphically. The numbers of tumorigenic cells in the blood or lungs of tumour-bearing mice were then found by comparing the survival times of bioassay recipients with survival times of the control mice injected with known numbers of viable tumour cells as described above.

*Lung colonization potential of melanoma and carcinoma cells*

Graded doses of melanoma or Lewis carcinoma cells were injected via a caudal vein and the numbers of pulmonary nodules determined 21 days later following post-mortem fixation of the lungs with 1 ml buffered formalin and examination under a dissecting microscope.

### Results

*Validity of bioassays*

The results of control experiments to validate bioassay procedures for viable melanoma and carcinoma cells are given in Tables I and II. Direct comparisons were made between the survival times of mice after injection of the two types of tumour cells mixed with other cells or tissues. For each dose/cell mixture, two or three experiments were made and survival data from these experiments were similar. For example, the survival times of groups of 10 mice injected i.p. with $10^5$ B16F10 cells only were $23.6 \pm 3.8$, $23.8 \pm 5.6$ and $22.7 \pm 2.9$ days. Survival times of groups of 10 mice injected with $10^5$ B16F10 cells plus carrier cells in three experiments were $19.9 \pm 1.7$, $20.0 \pm 3.1$ and $21.0 \pm 2.1$ days. The mean survival data of all experiments best fit lines of the form $1/\overline{T}d = I + m \log c$ where $1/\overline{T}d$ is the average inverse time of death in days following an injection of c cells. For B16F10 cells the lines for cells only, cells plus carrier and cells plus lung mince had the same ($P < 0.05$) slope (m) $9.27 \times 10^{-3} \pm 0.89 \times 10^{-3}$ and probably the same intercept I, zero. The repression coefficients for the 3 lines were 0.96, 0.99, and 0.99 respectively. The slope, intercept, and regression coefficient for cells plus blood plus carrier were $6.14 \times 10^{-3} \pm 0.51 \times 10^{-3}$, $16.6 \times 10^{-3} \pm 1.98 \times 10^{-3}$ and 0.99 respectively. There were no significant differences between lines calculated as above for the Lewis lung carcinoma groups shown in Table II. The pooled slopes, intercepts and regression coefficients were $1.06 \times 10^{-2} \pm 0.07 \times 10^{-2}$, $2.12 \times 10^{-2} \pm 0.27 \times 10^{-2}$ and 0.99 respectively.

The use of survival times to estimate the number of tumorigenic cells is a common application of this

**Table I** Bioassay of B16F10 melanoma cells

| *No. cells injected i.p.* | *Mean survival time [days ± s.d. (no. mice)]* B16F10 only | B16F10 + carrier cells[a] | B16F10 + lung mince[b] | B10F10 + blood[c] + carrier cells |
|---|---|---|---|---|
| $10^6$ | 17.3 ± 2.5 (10/10) | 15.9 ± 1.6 (10/10) | 16.1 ± 2.2 (10/10) | 19.0 ± 2.9 (10/10) |
| $10^5$ | 22.7 ± 2.9 (10/10) | 19.9 ± 1.7 (10/10) | 19.8 ± 1.1 (10/10) | 20.2 ± 3.1 (10/10) |
| $10^4$ | 28.3 ± 1.2 (10/10) | 25.1 ± 4.4 (10/10) | 26.1 ± 1.2 (10/10) | 25.3 ± 3.7 (10/10) |
| $10^3$ | 39.3 ± 1.7 (4/10) | 34.6 ± 8.2 (10/10) | 35.4 ± 1.2 (10/10) | 34.7 ± 11.5 (10/10) |
| $10^2$ | 39 (1/9) | 49.1 ± 7.9 (9/10) | 47.6 ± 2.3 (10/10) | 39.0 ± 7.8 (8/10) |
| $10^1$ | — (0/10) | 63.8 ± 10.4 (5/10) | Not done | 59.7 ± 12.3 (3/10) |

[a]$10^6$ B16F10 cells exposed to 50 Gy gamma radiation.
[b]B16F10 cells were added to normal lungs and the mixture minced before injection.
[c]0.5 ml heparinized normal mouse blood.

**Table II** Bioassay of Lewis lung carcinoma cells

| *No. cells injected i.p.* | *Mean survival time [days ± s.d. (no of mice)]* Lewis carcinoma + carrier cells | Lewis carcinoma + lung mince | Lewis carcinoma + blood + carrier cells |
|---|---|---|---|
| $10^6$ | 12.9 ± 3.0 (10/10) | 12.0 ± 2.6 (5/5) | 11.0 ± 0.9 (10/10) |
| $10^5$ | 14.8 ± 3.2 (10/10) | 13.3 ± 1.9 (5/5) | 12.7 ± 1.4 (10/10) |
| $10^4$ | 18.0 ± 3.0 (10/10) | 16.0 ± 1.6 (5/5) | 15.5 ± 0.9 (10/10) |
| $10^3$ | 20.2 ± 2.8 (10/10) | 20.0 ± 1.6 (5/5) | 18.0 ± 0.9 (10/10) |
| $10^2$ | 22.5 ± 5.9 (6/10) | 26.0 ± 1.8 (4/5) | 25.5 ± 6.7 (10/10) |
| $10^1$ | 32 (1/10) | Not done | 31.4 ± 12.1 (5/10) |

type of regression line. Such a procedure does not permit estimation of a standard error due to the fact that standard errors are estimated from the variance in a sample population. The method used to determine regression curves assumes the number of cells inoculated to be exact. Therefore, there is no information available to determine s.e. but it is possible to calculate 95% confidence levels. In the units used in these experiments, the 95% confidence levels are approximately $\pm 1\ \log_{10}$ unit and the calculated "range" data shown in Tables III and IV take these confidence limits into account.

The data in Table I also clearly show that the addition of carrier cells, lung mince, and blood plus carrier to B16F10 cells enhanced the number of tumour takes at low cell inocula compared with the incidence of takes in mice given B16F10 cells only. For instance, $10^2$ B16F10 cells grew in only 1/9 mice but the incidence of growth from the same dose of cells mixed with carrier cells was 90% ($P<0.05$, chi$^2$ test), or with lung mince was 100% ($P<0.05$, chi$^2$ test) or with blood plus carrier cells was 80% ($P<0.05$, chi$^2$ test). Even at inocula of as few as 10 cells, significant numbers of mice developed tumours in the presence of "carrier" cells. Thus, the threshold sensitivity of the bioassays is in the range of 10–100 tumorigenic cells and such survival data can be used to determine the numbers of tumorigenic cancer cells in the lungs and blood of tumour-bearing mice.

*Tumorigenic cells in circulation and lungs of B16F10 melanoma-bearing and Lewis lung carcinoma-bearing mice*

The peripheral blood and lungs of groups of mice bearing i.m. B16F10 melanomas were bioassayed at intervals throughout i.m. tumour growth in two separate experiments and the results are given in Table III. In the second experiment the primary i.m. tumours did not achieve the same sizes as in the first experiment, even though the same numbers of cells ($10^5$) were initially inoculated and survival times were comparable (23.2 ± 2.9 and 25.7 ± 2.5 days respectively). Nevertheless, the results of the two experiments were essentially similar in that progressively more animals had tumorigenic cells in their circulation and/or lungs as the i.m. tumour reached lethal proportions.

Two series of bioassay experiments were also

**Table III** Tumorigenic cells in circulation and lungs of B16F10 melanoma-bearing mice

| *Tumour age (days)* | *Tumour vol. (±s.d.)* | *Tumorigenic cells in lungs* Median | Range | Incidence | *Tumorigenic cells in blood*[a] Median | Range | Incidence | *Pulmonary metastasis* |
|---|---|---|---|---|---|---|---|---|
| Expt. 1 | | | | | | | | |
| 4 | <1.0 | * | * | 0/10 | * | *−<100 | 1/10 | 0/10 |
| 7 | <1.0 | * | * | 0/10 | * | * | 0/10 | 0/10 |
| 11 | 1.9±0.9 | <100 | *−$10^4$ | 5/10 | * | *−<100 | 1/10 | 0/10 |
| 14 | Not done | <100 | *−$10^4$ | 7/10 | * | *−<100 | 2/10 | 0/10 |
| 18 | 6.0±1.8 | 500 | *−$5\times10^6$ | 8/10 | <100 | *−$3\times10^3$ | 5/10 | 3/10 |
| 20 | 8.5±1.3 | 500 | *−$5\times10^6$ | 9/10 | * | *−$10^4$ | 4/10 | 3/10 |
| 22 | 10.0±1.3 | $10^3$ | <100−$5\times10^6$ | 15/15 | $5\times10^2$ | *−$10^4$ | 9/15 | 11/15 |
| Expt. 2 | | | | | | | | |
| 4 | <0.1 | * | * | 0/10 | * | * | 0/10 | 0/10 |
| 7 | 0.1±0.04 | * | * | 0/10 | * | * | 0/10 | 0/10 |
| 11 | 0.5±0.3 | * | *−$3\times10^3$ | 3/10 | * | *−$3\times10^3$ | 1/10 | 0/10 |
| 14 | 1.4±0.9 | 500 | *−$6\times10^3$ | 8/10 | * | *−$10^4$ | 4/10 | 0/10 |
| 18 | 3.2±0.9 | $2\times10^3$ | *−$8\times10^4$ | 9/10 | $6\times10^2$ | *−$7\times10^4$ | 5/10 | 0/10 |
| 21 | 5.5±1.6 | $5\times10^3$ | 800−$8\times10^4$ | 10/10 | $6\times10^3$ | *−$2\times10^4$ | 8/10 | 0/10 |

[a]0.5 ml.
*None detectable.

**Table IV** Tumorogenic cells in circulation and lungs of Lewis lung carcinoma-bearing mice

| *Tumour age (days)* | *Tumour vol. (±s.d.)* | *Tumorigenic cells in lungs* Median | Range | Incidence | *Tumorigenic cells in blood*[a] Median | Range | Incidence |
|---|---|---|---|---|---|---|---|
| Expt. 1 | | | | | | | |
| 4 | <0.1 | * | * | 0/10 | * | * | 0/10 |
| 7 | <0.1 | * | * | 0/10 | * | * | 0/10 |
| 11 | 0.3±0.1 | * | *−$10^3$ | 4/10 | * | *−$3\times10^3$ | 3/10 |
| 14 | 1.2±0.5 | $10^4$ | 400−$10^5$ | 10/10 | $10^3$ | *−$5\times10^4$ | 8/10 |
| 18 | 2.1±0.6 | $4\times10^5$ | 400−$10^6$ | 9/10 | $10^4$ | 400−$5\times10^4$ | 9/10 |
| 21 | 2.9±0.8 | $10^6$ | $10^4$−$3\times10^6$ | 10/10 | $5\times10^4$ | $7\times10^3$−$3\times10^6$ | 10/10 |
| 25 | 3.8±0.8 | $3\times10^6$ | *−$5\times10^7$ | 9/10 | $10^4$ | $10^3$−$5\times10^4$ | 9/10 |
| Expt. 2 | | | | | | | |
| 4 | <0.1 | * | * | 0/10 | * | *−<100 | 1/10 |
| 7 | <0.1 | * | * | 0/10 | * | *−<100 | 1/10 |
| 11 | <0.1 | * | * | 0/8 | * | * | 0/8 |
| 14 | 0.1±0.2 | <100 | *−400 | 5/10 | * | *−800 | 3/10 |
| 18 | 0.7±0.6 | $10^3$ | *−$3\times10^3$ | 6/9 | * | *−800 | 3/9 |
| 21 | 1.2±0.8 | $7\times10^3$ | *−$4\times10^5$ | 8/9 | <100 | *−$5\times10^4$ | 5/9 |
| 25 | 2.1±1.1 | $7\times10^4$ | *−$4\times10^5$ | 8/10 | <100 | *−$3\times10^3$ | 5/10 |
| 28 | 2.9±1.1 | $5\times10^4$ | *−$10^6$ | 7/10 | $10^4$ | *−$5\times10^4$ | 7/10 |

[a]0.5 ml.
*None detectable.

made with mice bearing i.m. Lewis lung carcinomas and the results are presented in Table IV. In the second experiment, the i.m. tumours grew at a slower rate. Survival times of mice given $10^5$ Lewis cells i.m. were 24.2±2.2 and 31.8±8.9 days in the first and second experiments respectively, even though the survival times of mice injected i.p. with $10^5$ cells from the same suspension used for i.m. injections were similar, 15.4±2.1 and 14.8±3.2 days respectively. Again, there were wide variations in the numbers of tumour cells detected in individual mice, even in those from mice with tumours of similar age and diameter.

The presence of cancer cells in the circulation of mice bearing B16F10 melanomas or Lewis lung carcinomas did not necessarily indicate that the same mice also had tumorigenic cells in their lungs, or vice versa. From Day 11 throughout tumour growth ~40% of B16F10 melanoma-bearing mice had tumorigenic cancer cells in both blood and lungs, but the incidence of melanoma-bearing mice with melanoma cells in their blood but not lungs was low (2%). Similarly, 60% of Lewis carcinoma-bearers had tumorigenic cells in both the circulation and lungs but <2% had cancer cells in their blood only. Forty percent of melanoma-bearing mice and 15% of carcinoma-bearers had no detectable circulating tumorigenic cells but did have tumorigenic cells in their lungs. Similar proportions of (20–25%) of melanoma- and Lewis carcinoma-bearing animals had neither circulating nor tumorigenic cells at the time of assay. An interesting finding was that in the presence of circulating cancer cells, 98% of animals had tumorigenic cells in their lungs.

To examine the possibility that cancer cells appearing in the blood or lungs during the first few days of tumour growth were the result of inadvertent introduction of tumour cells into the circulation during the initial i.m. injections, experiments were performed in which blood and lungs of groups of 5 mice were bioassayed immediately, 1 h and 4 h after i.m. injections of Lewis lung carcinoma cells. No cancer cells were detected in blood or lungs of these mice.

*Lung colonization potential*

The results in Table V show that, following injection of graded doses of cancer cells directly into the circulation, between $10^3$ and $10^4$ melanoma cells were needed to induce pulmonary nodules in 100% of mice and between $10^5$ and $10^6$ carcinoma cells were needed to give the same incidence.

Experiments were also made to determine the tumorigenicity of cancer cells shortly after their arrest in the lungs. Groups of 5–10 mice were given $10^5$ B16F10 melanoma cells via a tail vein. Five minutes later, their lungs were removed for bioassay and the results showed that the median numbers of tumorigenic cells were only $7 \times 10^3$ and $8 \times 10^3$ in duplicate experiments. Two experiments using Lewis lung carcinoma cells showed that the numbers of tumorigenic cells were also rapidly reduced to a similar extent within five minutes of injection, from $10^6$ originally injected to $6 \times 10^4$ and $7.5 \times 10^4$ in the two experiments.

### Discussion

In common with earlier attempts to quantify release of cancer cells from primary lesions by the use of perfusion systems (Liotta *et al.*, 1974; Butler & Gullino, 1975), experiments involving direct counting of circulating cancer cells (Glaves, 1983) also give information about the influx of malignant cells into the blood. However, the bioassay system developed in the present studies provides a measure of the biologically critical property of these disseminated cancer cells, their potential tumorigenicity.

Bioassays have been used previously to detect otherwise covert cancer cells in tissues and body fluids (Koike *et al.*, 1964; Grazet, 1966; Donelli *et al.*, 1969; Wexler *et al.*, 1971). However, in contrast to the present study, these former assays were often used to determine presence or absence of tumorigenic cells with few attempts (Döbrössy & Turi, 1976 Nakadate *et al.*, 1979) to define the limits of sensitivity of the assay or to obtain precise quantification of tumour cell load throughout tumour growth. The limiting dose bioassay method used here indicates that there is an inherent inefficiency of tumour development for both cell types which is possibly a reflection of the various host defense reactions mounted by the host against

**Table V** Pulmonary colonization potential of B16F10 melanoma and Lewis lung carcinoma cells following i.v. injection

| *No. cells injected* | *Pulmonary nodules*[a] *B16F10 melanoma median* | *range* | *incidence* | *Lewis lung carcinoma median* | *range* | *incidence* |
|---|---|---|---|---|---|---|
| $10^6$ | | —[b] | | 32 | 19–33 | 4/4 |
| $10^5$ | >500[c] | — | 5/5 | 1 | 0–2 | 4/5 |
| $10^4$ | 61 | 57–96 | 5/5 | 0 | 0–9 | 2/5 |
| $10^3$ | 6 | 2–10 | 5/5 | 0 | 0–1 | 1/5 |
| $10^2$ | 2 | 0–2 | 3/5 | | not done | |

[a]21 days after i.v. inoculations.
[b]mice died immediately after injection.
[c]coalesced nodules, too numerous to count.

malignant cells (Hanna, 1980; Goldfarb & Herberman, 1982). However, by taking advantage of the growth-promoting effects of lethally irradiated cells (Révész, 1955), the threshold dose is reduced to 10–100 cells.

In many experimental studies of chemotherapy regimens, lymphoid tumours such as the L1210 are used because the numbers of cells surviving after treatment can be accurately quantitated from survival curves (Skipper *et al.*, 1964, 1965) similar to those used in the present studies. These earlier studies relied upon the fact that these tumours will grow reproducibly from doses of $<10$ cells without growth-promoting manipulations. The bioassay developed here, however, will permit examinations of the effects of reduction therapy to be extended to solid tumours since inclusion of irradiated cells reduces the limits of quantitative detection to similarly low levels.

In the present studies, systematic bioassays showed that the entry of potentially tumorigenic cells into the circulation can be an early event, sometimes occurring within 4 days of tumour inoculation. A previous report (Stackpole, 1981) has indicated that cancer cells may be accidentally disseminated during inoculation into i.m. or other extravascular sites. However, the present bioassays detected no iatrogenically disseminated cells.

Although the numbers of tumorigenic cells in the circulation increased progressively during tumour growth, there were differences in the numbers of circulating tumorigenic cells released from tumours of the same age in duplicate experiments with the same tumour. These differences were associated with the growth rate of the intramuscular lesion rather than solely tumour size and it is therefore interesting to note that *in vitro* experiments have demonstrated that the faster their growth rate, the more easily cells may be detached from their substrates (Weiss, 1964). There were also wide fluctuations in the numbers of melanoma and carcinoma cells in both the blood and lungs of individual mice bearing tumours growing at the same rate. Such variations were also apparent in a study of circulating mouse lymphoma cells (Schirrmacher & Waller, 1982). It is possible that the rates at which cancer cells are released from a primary tumour could be a characteristic property of individual tumours. However, as previously suggested (Glaves, 1983), an alternative explanation is that these fluctuations reflect sporadic release of showers of cancer cells into the circulation so that samples taken over short periods of approximately one minute will necessarily contain variable numbers of cells depending upon the rate at which cancer cells are being released at that time.

The increases in circulating tumorigenic cells during primary tumour growth were paralleled by progressive accumulations of tumorigenic cells in the lungs. These cancer cells represent not only those cells immediately shed from the primary tumour, but also the progeny of earlier seedings. However, the experiments with the B16F10 melanoma indicate that pulmonary seeding of viable cancer cells resulted in as many as ten thousand potentially tumorigenic cancer cells in the lungs, yet no overt metastases are detectable at this time. Similarly, in experiments in which Lewis carcinoma cells were identified morphologically, only occasional pulmonary metastases were recorded until late in primary tumour growth, yet orders of magnitude more carcinoma cells had been seeded into the lungs by this time and most of these were tumorigenic on bioassay. At least $10^2$ B16F10 cells were needed to generate pulmonary nodules in a proportion of mice, even though this melanoma variant was selected for high lung colonization capacity (Fidler, 1973) and $10^3$ Lewis carcinoma cells were needed to generate at least one pulmonary lesion. Nevertheless, the maximum lung colonization potential of either tumour is not realized during spontaneous metastasis. It has been known for many years that relatively few cancer cells of any type produce overt lesions following direct injection into the circulation and, in common with the present studies, in those investigations in which circulating cancer cells were shed spontaneously from solid tumours, either metastases did not occur (Butler & Gullino, 1975) or were orders of magnitude less than the numbers of cancer cells released from the primary lesion (Liotta *et al.*, 1974). This dose-response relationship has recently been described as a contribution to "metastatic inefficiency" (Weiss, 1982). The present study shows that this inefficiency is not related to the tumorigenic potential of cancer cells leaving the primary tumour. This emphasizes the importance of those active specific and non-specific defense processes (Weiss & Glaves, 1976; Fidler *et al.*, 1977; Riccardi *et al.*, 1979; Glaves, 1980) operating in the lungs which contribute to the death of as many as 98% of these two types of tumour cells within 24 h of their initial arrest (Glaves, 1983; Fidler *et al.*, 1976). These previous reports describe the fate of radiolabelled tumour cells retained in the lungs after i.v. injections. Whilst the radiolabel used is associated with intact and potentially viable cells, the present experiments show that the vast majority of tumour cells are rendered non-tumorigenic within minutes of their arrest in the lungs. However, even though most of the seeded cells may die, the bioassays indicate that there is a considerable excess of tumorigenic doses of cells in the lungs at many times. These cells could represent a reservoir for secondary or tertiary metastases other organs and/or further overt metastases if the host had not died as a consequence of the primary tumour.

The numbers of circulating Lewis carcinoma cells quantified by bioassay were within similar ranges to those quantitated previously by direct counting techniques (Glaves, 1983) which indicates firstly, that the direct counting can be a reliable method for use where bioassays are impracticable. Secondly, the majority of cancer cells released into the venous circulation from intramuscular tumours are viable and at least potentially capable of generating secondary lesions. Independently and in concert, these two observations can offer valuable information about the quantitative and temporal aspects of rate-limiting stages of the post-invasive phases of metastasis. It will now be possible to examine those host- or tumour-associated factors which can potentially contribute to the release of cancer cells from primary tumours. For example, *in vitro* studies indicate that proteases may play an important role in invasion of tissues or blood vessels (Strauli *et al.*, 1980), but this role can now be quantitatively assessed directly, *in vivo,* during spontaneous metastasis.

The therapy of metastatic disease is a particular problem in cancer patients, and experimental models in mice which could monitor the efficacy of treatment would be a valuable asset to the development of new treatment regimens, especially where solid tumours and sub-clinical micro-metastases or dormant cancer cells are involved. The present studies emphasize the potential magnitude of such sub-clinical populations of cancer cells in distant organs and the bioassay technique developed here offers a very sensitive and quantitative method of monitoring the clinically relevant property of these cells – their potential tumorigenicity. Experiments are now in progress to determine at which of the sequential steps of the metastasis cascade anti-metastatic agents operate, since the effects of therapy upon the primary tumour, disseminating cells, subclinical populations of cancer cells and overt metastases can now be dissociated using the present experimental systems.

The excellent technical assistance of Deborah Ketch and Carol Bastian is appreciated and the helpful criticisms of Dr Leonard Weiss are acknowledged. Assistance with statistical evaluations from Dr James Harlos is also appreciated.

Funding for this research project was provided by Grant No. CA 28362 and partly by Core Grant no. 5P30-CA16056 from the National Institutes of Health.

### References

BUTLER, T.P. & GULLINO, P.M. (1975). Quantitation of cell shedding into efferent blood of mammary adenocarcinoma. *Cancer Res.*, **35,** 512.

DÖBRÖSSY, L. & TÚRI, G. (1976). Bioassay of blood-born tumour cells on *in vivo* and *in vitro* systems. Acta Morph. *Acad. Sci* (*Hung.*), **24,** 331.

DONELLI, M.G., ROSSO, R. & GARATTINI, S. (1969). Quantitative studies on cancer dissemination. *Cancer Res.*, **29,** 414.

FIDLER, I.J. (1973). Selection of successive tumor lines for metastasis. *Nature,* **242,** 148.

FIDLER, I.J., GERSTEN, D.M. & BUDMEN, M.B. (1976). Characterization *in vivo* and *in vitro* of tumor cells selected for resistance to syngeneic lymphocyte-mediated cytotoxicity. *Cancer Res.*, **36,** 3160.

FIDLER, I.J., GERSTEN, D.M. & RIGGS, C.W. (1977). Relationship of host immune status to tumor cell arrest, distribution and survival in experimental metastasis. *Cancer,* **40,** 46.

GAZET, J.-C. (1966). The detection of viable circulating cancer cells. *Acta Cytol.*, **10,** 119.

GLAVES, D. (1980). Metastasis: reticuloendothelial system and organ retention of disseminated malignant cells. *Int. J. Cancer,* **26,** 115.

GLAVES, D. (1983). Correlation between circulating cancer cells and incidence of metastasis. *Br. J. Cancer,* **48,** 665.

GOLDFARB, R.H. & HERBERMAN, R.B. (1982). Characteristics of natural killer cells and possible mechanisms for their cytotoxic activity. *Adv. Inflamm. Res.*, **4,** 45.

HANNA, M.G. (1980). Macrophages in tumor immunity. *Adv. Exp. Med. Biol.*, **121,** 353.

KOIKE, A. (1964). Mechanism of blood-borne metastases. I. Some factors affecting lodgement and growth of tumor cells in the lungs. *Cancer,* **17,** 450.

LIOTTA, L.A., KLEINERMAN, J. & SAIDEL, G.M. (1974). Quantitative relationships of intravascular tumor cells, tumor vessels, and pulmonary metastases following tumor implantation. *Cancer Res.*, **34,** 997.

NAKADATE, T., SUZUKI, M. & SATO, H. (1979). Quantitative study on the liberation of tumor cells into the circulating blood. *Gann,* **70,** 435.

RÉVÉSZ, L. (1955). Effect of x-irradiation on the growth of the Ehrlich ascites tumor. *J. Natl Cancer Inst.*, **15,** 1691.

RICCARDI, C., PUCCETTI, P., SANTONI, A. & HERBERMAN, R.B. (1979). Rapid *in vivo* assay of mouse natural killer cell activity. *J. Natl Cancer Inst.*, **63,** 1041.

SCHIRRMACHER, V. & WALLER, C.A. (1982). Quantitative determination of disseminated tumor cells by $^{3}$H-thymidine incorporation *in vitro* and by agar colony formation. *Cancer Res.*, **42,** 660.

SKIPPER, H.E., SCHABEL, F.M. Jr. & WILCOX, W.S. (1964). Experimental evaluation of potential anticancer agents. XIII. On the criteria and kinetics associated with "curability" of experimental leukemia. *Cancer Chemo. Rep.*, **35,** 1.

SKIPPER, H.E., SCHABEL, F.M. Jr., WILCOX, W.S., LASTER, W.R., TRADER, M.W. & THOMPSON, S.A. (1965). Experimental evaluation of potential anticancer agents. XVIII. Effects of therapy on viability and rate of proliferation of leukemic cells in various anatomic sites. *Cancer Chemo. Rep.*, **47**, 41.

STACKPOLE, C.W. (1981). Distinct lung-colonizing and lung-metastasizing populations in B16 mouse melanomas. *Nature*, **289**, 789.

STRAULI, P., BARRETT, A.J. & BAICI, A. (1980). *Proteinases and Tumor Invasion*. New York: Raven Press, p. 00.

SUGIURA, K. & STOCK, C.C. (1955). Studies in a tumor spectrum. III. The effect of phosphoramide on the growth of a variety of mouse and rat tumors. *Cancer Res.*, **15**, 38.

WEISS, L. (1964). Studies on cellular adhesion in tissue culture. VII. Surface activity and cell detachment. *Exptl. Cell Res.*, **33**, 277.

WEISS, L. (1982). Metastatic inefficiency. In: *Liver Metastasis*, (Eds. Weiss & Gilbert), Boston: G.K. Hall, p. 126.

WEISS, L. & GLAVES, D. (1976). The immunospecificity of altered arrest patterns of circulating cancer cells in tumor-bearing mice. *Int. J. Cancer*, **18**, 774.

WEISS, L., MAYHEW, E., RAPP, D.G. & HOLMES, J.C. (1982). Metastatic inefficiency in mice bearing B16 melanomas. *Br. J. Cancer*, **45**, 44.

WEXLER, H., KENADY, D.E. & KETCHAM, A.S. (1971). Bioassay of circulating murine tumor cells: A comparison between intravenous and intramuscular inoculation on tumor incidence. *Cancer*, **27**, 56.